# The clinical value of screening for acetaminophen in all patients with intentional overdose or altered mental status suspected to be secondary to overdose

**DOI:** 10.3389/fphar.2025.1633548

**Published:** 2025-07-30

**Authors:** Bader Alyahya, Abdulaziz Alalshaikh, Mohammed Almohawes, Mosaed Alnowiser, Omar Alsuliman, Rand Alrefaei, Sarah Alaidarous, Maha Alnahdi, Shadi Tamur, Musa Alfaifi, Mohammed Al Deeb, Zohair A. Al Aseri

**Affiliations:** ^1^ Emergency Medicine Department, College of Medicine, King Saud University, Riyadh, Saudi Arabia; ^2^ College of Medicine, King Saud University, Riyadh, Saudi Arabia; ^3^ Department of Pediatrics, College of Medicine, Taif University, Taif, Saudi Arabia; ^4^ Department of Emergency Medicine, Armed Forces Hospital, Khamis Mushait, Saudi Arabia; ^5^ Department of Emergency Medicine, College of Medicine, King Saud Bin Abdulaziz University for Health Sciences, Riyadh, Saudi Arabia; ^6^ Department of Clinical Sciences, College of Medicine and Riyadh Hospital, Dar Al Uloom University, Riyadh, Saudi Arabia; ^7^ Adult Critical Care, Therapeutic Deputyship, Ministry of Health, Riyadh, Saudi Arabia

**Keywords:** acetaminophen level, acetaminophen toxicity, Saudi Arabia, acetaminophen screening, silent paracetamol overdose

## Abstract

**Background:**

Acetaminophen (APAP) is commonly coingested in cases of suicide or intoxication because it is widely available, effectively analgesic and antipyretic, and it is often combined with other medications, such as opioids and antihistamines. APAP overdose often causes no symptoms or nonspecific symptoms in the first 12–24 h after ingestion. Delayed diagnosis is associated with a reduced response to antidote and sometimes liver failure and mortality. However, ordering unnecessary test is not cost-effective specially if it is mostly negative, and empirical therapy is associated with significant cost and possible adverse effects.

**Methods:**

This single-center retrospective study was conducted at King Saud University Medical City (KSUMC) in Riyadh, Saudi Arabia. Our population included all patients who presented to the emergency department with intentional drug overdose or altered mental status (AMS) suspected to be related to an overdose between June 2015 and January 2024 but with a history that was not suggestive of APAP overdose. Medical records were reviewed for patient information on demographic data, overdose details, and documentation of the clinical features of toxicity. All the subjects were kept anonymous; we used code numbers as identifiers. The data collected by the investigators were entered into an Excel worksheet in an encrypted format.

**Results:**

A total of 2914 patients were screened for acetaminophen (APAP) levels and 1517 met our inclusion criteria. Fourteen (0.9%) patients had detectable levels (>10 μg/mL) despite a negative history. Three (0.2%) patients had levels above 100 μg/mL and were treated with N-acetylcysteine (NAC).

**Conclusion:**

Our study revealed that a small number of patients who presented with intentional overdose but denied APAP ingestion or AMS suspected to be due to overdose had a positive APAP level. But, Given the serious consequences of APAP toxicity we cannot recommend stopping the screening for APAP specially in high-risk suicidal patients. A larger multicenter study is recommended to identify those high-risk patients. We also found deviation from the current guidelines regarding NAC administration in patients with positive APAP level when the time of ingestion is unknown.

## 1 Introduction

Acetaminophen (APAP) overdose has been recognized as one of the most common toxicities in Saudi Arabia and worldwide (Alghafees et al., 2022; Chidiac et al., 2023). Easy accessibility, over-the-counter availability, and low cost are some of the main factors leading to this issue. APAP is widely used as an effective analgesic and antipyretic agent (Chidiac et al., 2023). APAP toxicity has been identified as a major cause of fulminant liver failure. Despite being considered a safe medication, APAP is often used for self-harm and attempted suicide (Yoon et al., 2016).

Diagnosing APAP toxicity heavily relies on the patient’s history and serum concentration, as the initial presentation may be vague, and patients are often asymptomatic in the first 24 h (Rowden et al., 2006). Owing to the fear of its serious complications, many physicians acquire a serum level, even if the patient denies taking it (Walls et al., 2022). A number of studies have reported that hardly any patient to have a positive acetaminophen level without any suggestive history among those presenting with intentional drug overdose or an altered mental status (AMS) likely related to poisoning (Bentur et al., 2011; Ashbourne et al., 1989; Sporer and Khayam-Bashi, 1996; Ala et al., 2019; Dargan et al., 2001; Hartington et al., 2002; Eldridge and Holstege, 2006). However, many of these studies recommend the use of an APAP level in that population given its low cost and the significant consequences of liver toxicity and, in some cases, mortality (Ashbourne et al., 1989; Hartington et al., 2002; Eldridge, 2007; Graham et al., 2006). However, the test is not always available and sometimes physicians request the test but then do not follow the existing consensus guidelines regarding treatment (Dart et al., 2023). Also, in peripheral hospitals, when APAP screening is not available, patients may need to be transferred a long distance just to obtain an APAP level or be treated empirically.

The necessity of routine measurement of APAP levels in patients with intentional drug overdose or AMS is a topic of debate in the medical community. Some studies suggest that routine measurement of APAP levels in these patients may not significantly affect the outcome (Ala et al., 2019; Dargan et al., 2001). Several studies have explored the issue of unnecessary ordering of diagnostic tests in various healthcare settings and studied emergency physicians’ perceptions of medically unnecessary investigations. They reported that physicians often order these tests because of concerns about malpractice and patient satisfaction rather than clinical necessity (Alalshaikh et al., 2022; Kanzaria et al., 2015).

An extensive literature review revealed no studies in our region evaluating the need for APAP level in patients with intentional drug overdose who denied APAP ingestion or those with AMS (therefore cannot provide history) suspected to be secondary to a drug overdose.

## 2 Methods

We conducted a single-center retrospective observational study at King Saud University Medical City (KSUMC) in Riyadh, Saudi Arabia, reviewing 2914 cases from 1 June 2015, to 1 January 2024, as our electronic health record system (Esihi) was implemented in 2015. This study assessed the clinical implications of APAP serum levels in patients presenting to the emergency department with suspected drug overdose. The study included all patients whose APAP level was ordered for them and then categorized into individuals who either denied APAP ingestion or those whose ingestion status was uncertain due to AMS or unreliable histories.

Patients under 14 years of age (as patients 14 years and above are seen in the adult emergency department), documented accidental APAP ingestion, those admitted to taking an APAP overdose, patients who received IV APAP in the hospital, or cases where documentation was inadequate were excluded.

We categorized APAP serum levels into two thresholds: a serum concentration of more than 10 μg/mL was considered a positive level (Dart et al., 2023), and a very high level was defined as any level exceeding 100 μg/mL.

Data were extracted from electronic health records and included whether N-acetylcysteine (NAC) was administered during the emergency visit and focused on patient demographics, overdose specifics, APAP serum levels, and liver toxicity markers, including transaminases and PT/INR levels.

To maintain subject anonymity, we used code numbers as identifiers, and all collected data were stored in an encrypted Excel sheet accessible only to the authors. This ensured strict confidentiality throughout the data extraction and analysis process.

### 2.1 Statistical analysis

The data were extracted and revised in an Excel sheet. Statistical analysis was conducted via the Statistical Program for Social Sciences (SPSS) computer program (version 26.0; Armonk, NY, United States). Categorical variables are presented as numbers and percentages. Continuous, nonnormally distributed variables are reported as the means (SDs), medians, and interquartile ranges (IQRs). The chi-square test and Fisher’s exact test were used to compare categorical variables. P-values less than 0.05 were considered statistically significant.

## 3 Results

**FIGURE 1:**
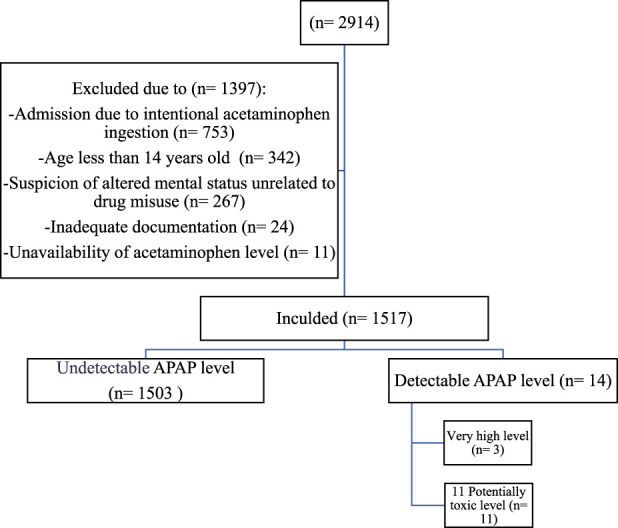
Study Flowchart. Abbreviations: AMS: Altered mental status; APAP: N-acetyl-p-aminophenol (acetaminophen).

As shown in Figure 1, 2914 patients presented to the ED of KSUMC between 1 June 2015, and 1 January 2024, and had APAP level measured. Of these, 1397 did not meet the inclusion criteria and were excluded.

A total of 1517 patients underwent acetaminophen (APAP) level screening and met our inclusion criteria. Males constituted 51.7% of the population. The median (IQR) age was 26 years. Approximately 42.5% of the studied patients had a history of psychiatric illness, whereas 23.9% had a history of street drug use. Notably, 8.5% patients had both a history of psychiatric illness and street drug use. Most of the included patients (84.5%) presented with intentional overdose but denied APAP ingestion, and the remainder presented with AMS suspected to be secondary to an overdose or poisoning. (Table 1).

**TABLE 1:** Demographics and clinical characteristics of patients with APAP levels.

Variables		Number	Percentage
Sex	Male	785	51.7
	Female	732	48.3
Age	Mean (SD)	29.98 (13.177)	
	Median (IQR)	26 (15)	
Marital status	Single	1122	74
	Married	395	26
History of psychological illness	Yes	645	42.5
	No	872	57.5
Type of psychological illness	Depression	293	19.3
	Psychosis	232	15.3
	Bipolar	70	4.6
	Anxiety	50	3.3
Previous suicide attempt	Yes	258	17
	No	1259	83
History of Street Drug Use	Yes	362	23.9
	No	1155	76.1
Type of street drug used	Amphetamine	152	10
	Benzodiazepines	97	6.4
	Pregabalin	73	4.8
	Cocaine	30	1.9
	Not documented	12	0.8
Presentation	Suspected or confirmed intentional overdose with negative history of APAP ingestion	1282	84.5
	AMS	235	15.5

Abbreviations: AMS: altered mental status; APAP: N-acetyl-p-aminophenol (acetaminophen).

Most included patients presented with negative APAP levels ≤10 μg/mL (n = 1503, 99.1%). Fourteen patients had positive APAP levels; eleven patients (0.7%) had APAP levels between 11 and 46.5 μg/mL, whereas three patients (0.2%) had very high levels exceeding 100 μg/mL. One patient presented with a level of >300 μg/mL and one had a level of 151 μg/mL, both are considered very high and fall above the treatment threshold regardless of the time of ingestion. Patients with APAP levels >100 μg/mL (n = 3, 0.2%) received NAC treatment (Table 2). Table 3 summarizes the clinical characteristics of three individuals with markedly elevated acetaminophen (APAP) levels.

**TABLE 2:** APAP level at presentation and management (n = 1517).

Variables		Number	Percentage
APAP level (μg/mL)	Very high level, likely Toxic (>100)	3	0.2
	Potentially toxic (>10–60)	11	0.7
	Undetectable (≤10)	1503	99.1
N-acetylcysteine treatment	Yes	3	0.2
	No	1514	99.8

Abbreviation: APAP: N-acetyl-p-aminophenol (acetaminophen).

**TABLE 3:** Patient characteristics among individuals with markedly elevated APAP level.

Variables	Patient no. 1	Patient no. 2	Patient no. 3
Sex	Male	Female	Female
Age	17	14	23
History of psychological illness	No	No	Yes
History of Street Drug Use	No	No	No
Presentation	AMS	Suspected intentional overdose with negative history of APAP ingestion	Suspected intentional overdose with negative history of APAP ingestion
APAP level	>300	151	114.2

Abbreviations: AMS: altered mental status; APAP: N-acetyl-p-aminophenol (acetaminophen).

Patients with AMS who were not able to report APAP ingestion were more likely to present with detectable levels than were those who reported negative APAP ingestion (2.6% vs 0.6%, P = 0.013). No other factors, including age, sex, or history of having a psychiatric illness or street drug use, were significantly associated with detectable APAP levels (Table 4).

**TABLE 4:** Risk factors for having high APAP levels.

Variables		APAP level		Total	P Value
		Detectable (n = 14)	Undetectable (n = 1503)		
Age median (IQR)		23 (9)	26 (15)	1517	0.140
Sex	Female	7 (1.0%)	725 (99.0%)	732	0.895
	Male	7 (0.9%)	778 (99.1%)	785	
Weight (Kg)	Mean weight	74.5	76.2	1517	0.538
History of psychiatry illness	Yes	3 (0.5%)	642 (99.5%)	645	0.109
	No	11 (1.3%)	861 (98.7%)	872	
History of street drug use	Yes	5 (1.4%)	357 (98.6%)	362	0.342
	No	9 (0.8%)	1146 (99.2%)	1155	
Presentation	Suspected or confirmed intentional overdose with negative history of APAP ingestion	8 (0.6%)	1274 (99.4%)	1282	0.013
	AMS	6 (2.6%)	229 (97.4%)	235	

Abbreviations: AMS: altered mental status; APAP: N-acetyl-p-aminophenol (acetaminophen).

## 4 Discussion

Intentional drug overdose is a common presentation to emergency departments. Screening for acetaminophen overdose is often part of universal testing in all poisoned patients. APAP testing is not always available, and patients may need to be transferred to a different healthcare facility and sometimes to a different city to obtain the APAP level or receive empirical treatment. There is always a risk when transferring patients, especially at night, and there is also a significant cost associated with that (Hernandez-Boussard et al., 2017; Yu et al., 2022; Mueller et al., 2019). The main objective of this study was to assess the clinical value of ordering APAP levels in patients presenting with an intentional drug overdose when they deny APAP ingestion or those with AMS suspected to be secondary to a drug overdose.

Our results support the findings of previous studies on this topic, as only 0.9% of the studied population was found to have a positive APAP level (>10 μg/mL), and only three of the 1517 patients (0.19%) had very high APAP levels and were treated with NAC. However, based on the standard practice which is supported by a recent consensus guideline by Dart et al., all 14 patients should probably have undergone NAC treatment because the time of ingestion is uknown (Dart et al., 2023). Ashbourne and colleagues demonstrated that among 486 patients who presented to their urban hospital with intentional drug overdose, seven patients had unrecognized acetaminophen overdose. However, only one patient was potentially hepatotoxic level and needed to be treated with NAC (Ashbourne et al., 1989).

Lucanie et al. evaluated the incidence of APAP overdose among patients presenting with suicidal ingestion. Among the 320 documented patients who denied acetaminophen exposure, 23 patients had levels above 10 μg/mL. NAC was given to twelve patients, seven of whom were identified to have toxic levels. Meanwhile, the other five had non-toxic or uninterpretable levels (Lucanie et al., 2002). Dargan and colleagues screened 411 patients who had a history of either drug overdose (n = 296) or AMS (n = 115). They reported that four patients (3.5%) in the AMS group had positive levels and were treated with NAC. All those who denied APAP ingestion were not found to have detectable levels (136 of 296) (Dargan et al., 2001). In contrast, several other studies have failed to find any patient with a potentially toxic level (Sporer and Khayam-Bashi, 1996; Ala et al., 2019; Hartington et al., 2002; Graham et al., 2006; Chan et al., 1995; Garner, 1996).

Among the three patients with very high levels, patient no. 1 was a 17-year-old male known to have sickle cell disease on regular oral folic acid, hydroxyurea, and oral opioids. He was brought by an ambulance after his mother found him lying on the floor. She was uncertain whether he ingested any medication or not, and no history could be obtained from the altered patient. He was found to have an APAP level >300 μg/mL.

Among the other two patients, patient no. 2 was a 25-year-old female known to have general anxiety disorder and borderline personality disorder who presented to the emergency department with her brother following the ingestion of 20–30 pills of 5-6 different medications. She was able to recall two of these medications (esitalopram and lamotrigine). Later, she was found to have an APAP level of 114 μg/mL, and treatment with NAC was initiated. Patient no. 3 was a 14-year-old female with a history of previous suicidal ingestion. She was brought in by her father after he found her unconscious with wrist-cutting marks. She denied any history of ingestion. Her APAP level was found to be 151 μg/mL and received NAC treatment. None of the three patients developed acute liver injury during their hospital stay, and all three patients achieved good recovery.

Camilleri deduced in his study that a history of APAP overdose is moderately reliable and more reliable than a history of street drug misuse (Camill and eri, 2015). Despite the rare incidences of potentially toxic APAP levels, the majority of the studies mentioned above recommended screening all patients with suspected drug overdose regardless of whether the history was suggestive. Bentur et al. concluded that overdosed patients who deny both APAP and multidrug ingestion can be considered reliable for APAP history. Therefore, they proposed that routine APAP screening in patients who denied both APAP and multidrug ingestions might not be needed, especially in healthcare centers with limited resources or where the APAP level is not readily available (Bentur et al., 2011). However, in our study, two of the fourteen patients with positive results presented with single drug ingestion and denied APAP ingestion.

### 4.1 Limitations

This study has several limitations. First, it was conducted at a single center, which may limit the generalizability of the findings. Additionally, since the study was based in an urban healthcare center, the results may not accurately represent the situation in peripheral hospitals or the impact of patient transfers. This study’s retrospective design carries inherent limitations. Specifically, it is susceptible to biases related to data availability, accuracy, and potential selection bias.

### 4.2 Conclusion

Our study revealed that a small number of patients who presented with intentional overdose but denied APAP ingestion or AMS suspected to be due to overdose had a positive APAP level. But, Given the serious consequences of APAP toxicity we cannot recommend stopping the screening for APAP specially in high-risk suicidal patients. A larger multicenter study is recommended to identify those high-risk patients. We also found deviation from the current guidelines regarding NAC administration in patients with positive APAP level when the time of ingestion is unknown.

## Data Availability

The raw data supporting the conclusions of this article will be made available by the authors, without undue reservation.

## Abbreviations

APAP, N-acetyl-p-aminophenol (acetaminophen); AMS, Altered mental status; KSUMC, King Saud University Medical City; NAC, N-acetylcysteine; μg/mL, microgram/mL.
